# Evidence from GC-TRFLP that Bacterial Communities in Soil Are Lognormally Distributed

**DOI:** 10.1371/journal.pone.0002910

**Published:** 2008-08-06

**Authors:** James R. Doroghazi, Daniel H. Buckley

**Affiliations:** 1 Department of Microbiology, Cornell University, Ithaca, New York, United States of America; 2 Department of Crop and Soil Sciences, Cornell University, Ithaca, New York, United States of America; Centre National de la Recherche Scientifique, France

## Abstract

The Species Abundance Distribution (SAD) is a fundamental property of ecological communities and the form and formation of SADs have been examined for a wide range of communities including those of microorganisms. Progress in understanding microbial SADs, however, has been limited by the remarkable diversity and vast size of microbial communities. As a result, few microbial systems have been sampled with sufficient depth to generate reliable estimates of the community SAD. We have used a novel approach to characterize the SAD of bacterial communities by coupling genomic DNA fractionation with analysis of terminal restriction fragment length polymorphisms (GC-TRFLP). Examination of a soil microbial community through GC-TRFLP revealed 731 bacterial operational taxonomic units (OTUs) that followed a lognormal distribution. To recover the same 731 OTUs through analysis of DNA sequence data is estimated to require analysis of 86,264 16S rRNA sequences. The approach is examined and validated through construction and analysis of simulated microbial communities in silico. Additional simulations performed to assess the potential effects of PCR bias show that biased amplification can cause a community whose distribution follows a power-law function to appear lognormally distributed. We also show that TRFLP analysis, in contrast to GC-TRFLP, is not able to effectively distinguish between competing SAD models. Our analysis supports use of the lognormal as the null distribution for studying the SAD of bacterial communities as for plant and animal communities.

## Introduction

The hollow curve of a species abundance distribution which results when most species are rare and a few are abundant is one of the few ecological patterns exhibited by almost all communities. A range of factors such as birth, death, migration, niche adaptation, lifestyle, chance, resource partitioning, and history can all contribute to the formation of this characteristic hollow curve (see [1] for review). The universality of this phenomenon is worthy of study in its own right, but SADs can also be useful in making predictions about community properties such as diversity and migration [2].

While McGill *et al* [1] count at least 40 different models that have been used to describe species abundance distributions, the most commonly invoked models are those based on lognormal, geometric, power-law, and Fisher's log-series distributions. The lognormal distribution in particular has been observed to approximate species distributions in a wide range of communities (e.g. [3], [4]). One possible reason for the success of the lognormal, as put forth by both May [5] and MacArthur [6] is that the multiplicative combination of normally distributed factors would result in lognormally distributed species' abundances. The lognormal SAD could also arise from other processes, including niche-partitioning or as the limit of population dynamics [1], [7], [8], [9]. The ecological processes that create these patterns, however, remain nebulous and require further investigation.

Vast microbial communities can exist within a fairly small space and as such microbial communities provide a potential opportunity for experimental approaches to exploring the ecological factors that influence SADs. Obtaining accurate SAD data from a microbial community can be a daunting task, however, due to the remarkable complexity of these communities [10]. Previous estimates of the number of bacterial species present in soil vary widely, from 4×10^3^ to 8.3×10^6^ taxa in 10–30 g of soil representing around 10^8^–10^10^ individuals [11], [12]. Because we are currently unable to culture the vast majority of bacteria, clone libraries of 16S rRNA genes are generally used to characterize the structure and composition of microbial communities. Samples of several hundred 16S rRNA genes are common but fail to provide sufficient data to effectively distinguish between competing models of species abundance distributions [13], [14], [15], [16]. One estimate of the sampling required to encounter just half of the operational taxonomic units (OTUs) present in one g of soil ranges from 16,284 to 44,000 [13]. Dunbar *et al* [13] have examined the SAD of four different bacterial soil communities from Arizona by sequencing 16S rRNA genes from each community. They found that in 200 member surveys 93% of the species-level groups were present only once, which is suggestive of inadequate sampling, as pointed out by the authors [13]. Despite the sampling level, the authors found that the available data best followed a lognormal distribution. Likewise, Schloss and Handelsman [15] also found evidence that a truncated lognormal distribution provided the most consistent approximation of the microbial community in an Alaskan soil.

To assess the SAD of soil bacteria we have combined two common techniques, terminal restriction fragment length polymorphism (TRFLP) and density gradient centrifugation of community DNA. TRFLP is a community fingerprinting method in which diverse gene sequences are amplified by PCR using a fluorescently labeled primer. The DNA is then cut with a restriction enzyme and the sizes of the resulting mixture of labeled terminal restriction fragments (TRF) are determined through fragment analysis. Closely related individuals will typically generate a TRF of the same size, but in many cases unrelated individuals will share a TRF and this result causes a loss of information. Some of this information can be preserved by fractionating the DNA prior to TRFLP analysis. Genomic DNA can vary in mol% G+C content from 30% to 80% and can be separated on this basis due to differences in DNA buoyant density in a CsCl density gradient [17]. Holben et al. [17] have previously shown GC-DGGE to be useful for examining rare bacterial community members. Likewise, by using density-dependent fractionation of genomic DNA prior to TRFLP profiling (GC-TRFLP) it is possible to greatly increase the information available from the community by decreasing the number of overlapping TRFs from unrelated bacteria. One advantage of GC-TRFLP is that fragment separation is completely independent of DNA G+C content. In contrast, fragment separation in denaturing gradient gel electrophoresis is partly dependent on DNA fragment G+C content and this may reduce the potential resolution of GC-DGGE relative to GC-TRFLP. The advantage of either GC-DGGE or GC-TRFLP in relation to clone libraries is the ability to rapidly screen many taxa simultaneously and at greatly reduced cost. While the OTUs defined from TRF data are in no way comparable to bacterial species it should be noted that a similar limitation exists in the analysis of 16S rRNA gene sequences. It has been well documented that 16S rRNA sequence similarity cutoffs used to define operational taxonomic units are arbitrarily assigned and do not correspond to an ecologically meaningful species concept [18]. Regardless, the examination of discrete units of genetic diversity can help to provide insights on the factors that govern the structure of microbial communities.

Clearly any approach for assessing microbial community structure must be evaluated with respect to whether the sampling depth achieved is sufficient to provide useful information about the community distribution [10]. To evaluate the ability of the GC-TRFLP approach to accurately estimate community distributions we have created artificial communities with known species abundance distributions and subjected them to GC-TRFLP in silico. Distributions were estimated for GC-TRFLP data from real and simulated communities using an iterative algorithm that estimates distribution parameters by minimizing χ^2^ between observed and modeled data. This approach was also used to contrast the abilities of TRFLP and GC-TRFLP to recapitulate community SAD and to examine the potential impact of PCR bias on the accuracy of microbial community SAD estimation.

## Results

### GC-TRFLP of a soil community

**Figure 1:**
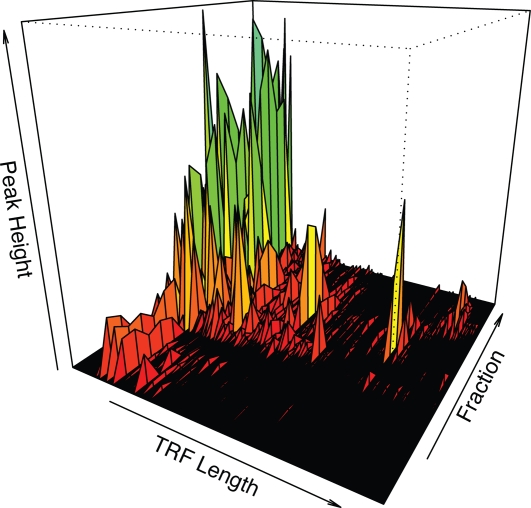
Plot of GC-TRFLP data for a soil sample indicating TRFLP results for CsCl gradient fractions containing DNA of different G+C content. Peaks are colored based on height to increase contrast between peaks.

**Figure 2:**
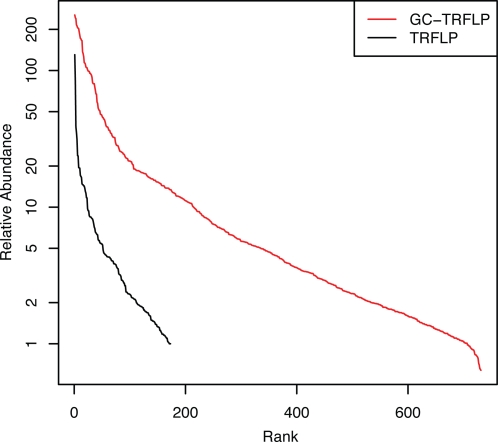
Rank abundance of both GC-TRF and TRF peak height values for GC-TRFLP and TRFLP performed on the same soil sample. The GC-TRFLP data represents 731 OTUs, while the TRFLP data represents 173 peaks.

**Figure 3:**
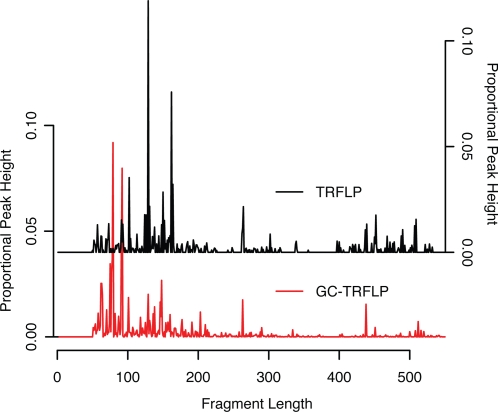
GC-TRFLP and TRFLP electropherograms plotted on offset baselines. The composite GC-TRFLP was created by collapsing the fraction axis, adding the peak height of identical TRFs across fractions. Peaks are represented as a proportion of total peak height.

**Figure 4:**
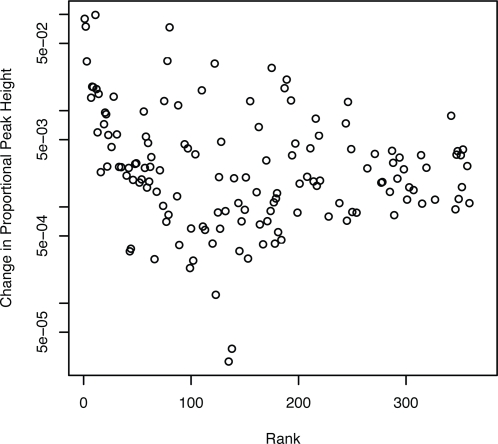
Comparison of differences in relative peak height between composite TRFs from GC-TRFLP and matching TRFs from TRFLP which shows that variation in peak height between TRFLP and GC-TRFLP increases with increasing peak height. Comparison is ordered based on peak rank in composite GC-TRFLP.

A single soil sample was subjected to both TRFLP and GC-TRFLP analysis of bacterial 16S rRNA genes. GC-TRFLP identified 731 OTUs, defined by genome G+C content and TRF size (Figures 1 and 2). In contrast, TRFLP analysis of the same DNA sample resulted in 173 discrete TRFs (Figures 2 and 3). To facilitate comparison with TRFLP, the TRFs from GC-TRFLP were composited without respect to genome G+C content and their cumulative peak heights summed (Figure 3). GC-TRFLP generated a total of 359 distinct TRFs demonstrating that GC-TRFLP enhanced recovery of TRFs not accessible through conventional TRFLP; as has been previously suggested for a similar method [17]. A total of 85.5% of the TRFs detected in TRFLP were also detected in GC-TRFLP (Figure 3). Variation between TRFLP and composite GC-TRFLP increased as a function of rank abundance suggesting a relationship between peak height and variance in peak height which might be expected for these data (Figure 4).

### Testing the ability of GC-TRFLP to estimate community SAD

To test the validity of estimating community SAD from GC-TRFLP data we have created a range of artificial communities by drawing sequences at random from a public database and assigning different abundance values to these sequences by using one of several known distributions. GC-TRFLP was performed in silico on these communities and the resulting GC-TRFLP data was used to estimate the SAD of the artificial communities. The results show that in every case the K-S test rejects all distributions except the one which matched the underlying distribution (Table 1). Due to the flexibility of the lognormal distribution, fitting with the lognormal was able to minimize the χ^2^ value for non-lognormally distributed communities almost as well as the distributions that were used to generate those communities (Table 1). For instance, the χ^2^ value for a lognormal distribution fit to a community created with a power-law distribution is 65.7, whereas the best fitting power-law estimate for that community gives a χ^2^ value of 60.1. Despite this flexibility, the D-statistic is clearly able to reject the lognormal in cases where it does not represent the true distribution of the community (Table 1). This result suggests that while the lognormal is flexible the K-S test has sufficient power to reject distributions derived from GC-TRFLP data when they do not accurately represent the underlying community.

**Table 1:** Evaluation of distributions that were fit to simulated GC-TRFLP of artificial communities when the original distributions of those communities were known.

Original	Estimate	χ^2^	D-statistic	*p*-value
Lognormal	Lognormal	63.1	0.031	0.826*
Power	Power	60.1	0.033	0.797*
Geometric	Geometric	62.8	0.033	0.776*
Fisher's	Fisher's	51.7	0.032	0.820*
Power	Lognormal	65.7	0.087	0.026
Geometric	Lognormal	113.8	0.124	0
Fisher's	Lognormal	132.8	0.216	0

* Indicates a failure to reject the null hypothesis that the two distributions are the same as determined by the KS test.

### Estimating the SAD of the soil community from GC-TRFLP

**Figure 5:**
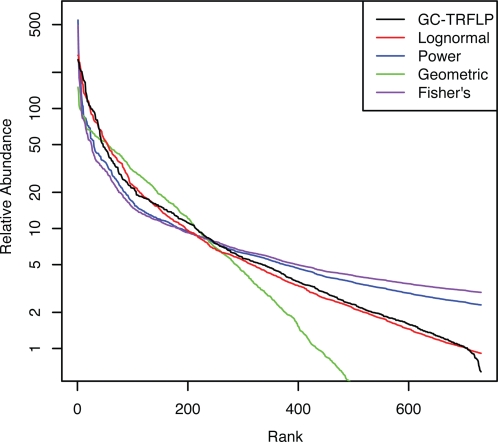
GC-TRFLP data for soil and the distributions that provided the best fit to this data. Goodness-of-fit measurements are given in Table 2.

**Figure 6:**
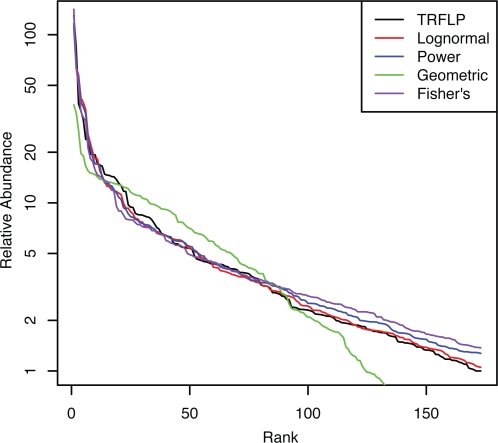
TRFLP data for soil and the distributions that provided the best fit to this data. Goodness-of-fit measurements are given in Table 3.

The lognormal distribution was found to provide the best fitting estimate to the GC-TRFLP data from soil (Table 2, Figure 5). The K-S test could not reject the null hypothesis that the lognormal estimate and the GC-TRFLP data were from the same distribution (*p*-value 0.657). In contrast, the K-S test rejected all other distributions tested (Table 2). As expected, data derived from TRFLP analysis had less ability to exclude potential distributions than GC-TRFLP (Figure 6). From the TRFLP data we could reject the geometric distribution and narrowly rejected Fisher's distribution, but could not distinguish between the power and lognormal distributions (Table 3).

**Table 2:** Evaluation of the fit of different distributions to GC-TRFLP data from the soil community.

Estimate	χ^2^	D-statistic	*p*-value	Parameters		
Lognormal	155.5	0.045	0.474*	α∶11850.7	μ∶4.27	σ∶2.01
Power	1807.5	0.323	0	α∶544.6	β∶−0.785	
Geometric	2126.581	0.369	0	α∶67.40	β∶0.0077	
Fisher's	2661.275	0.394	0	α∶0.119	β∶0.00003	

* Indicates a failure to reject the null hypothesis that the two distributions are the same as determined by the KS test.

**Table 3:** Evaluation of the fit of different distributions to TRFLP data from the soil community.

Estimate	χ^2^	D-statistic	*p*-value
Lognormal	288.85	0.076	0.686*
Power	280.25	0.111	0.289*
Geometric	2634.48	0.264	0
Fisher's	406.67	0.161	0.047

* Indicates a failure to reject the null hypothesis that the two distributions are the same as determined by the KS test.

### Evaluating the effect of PCR bias on SAD estimates

With exceptions [11], [19] nearly all methods used to asses bacterial SAD depend on the analysis of genes amplified by PCR. The potential for the relative abundance of gene sequences to change during PCR is well known but the systematic effect of PCR bias on efforts to evaluate community SAD have not previously been considered. To assess the possibility that PCR bias influences distribution fitting, we generated artificial communities of known distribution and applied a simple random bias of up to 38% to each sequence, a value chosen from the TRFLP PCR bias study performed by Frey et al [20]. GC-TRFLP was then performed on these samples in silico and the resulting data used to estimate community SAD (Table 4). The Fisher's log-series and power-law distributions were both erroneously rejected for communities assembled with each of these functions following application of PCR bias (Table 4). In contrast, the lognormal distribution could be rejected for communities assembled with either a Fisher's log-series or geometric distribution despite the PCR bias applied (Table 4). Most interestingly, the lognormal could not be rejected by the K-S test when a PCR bias was applied to a community having a power-law distribution (Table 4).

**Table 4:** Impact of potential PCR bias on estimates (See table 2).

Original	Estimate	χ^2^	D-statistic	*p*-value
Biased Lognormal	Lognormal	65.42	0.041	0.591*
Biased Power	Power	68.98	0.083	0.020
Biased Geometric	Geometric	87.43	0.041	0.587*
Biased Fisher's	Fisher's	63.56	0.092	0.005
Biased Power	Lognormal	60.13	0.065	0.125*
Biased Geometric	Lognormal	149.31	0.127	0
Biased Fisher's	Lognormal	113.50	0.151	0

* Indicates a failure to reject the null hypothesis that the two distributions are the same as determined by the KS test.

## Discussion

We have used GC-TRFLP to analyze the distribution of 731 bacterial OTUs from one gram of soil and found that the lognormal distribution provides the best fit to the data, and was the only distribution tested that could not be rejected as significantly different from the GC-TRFLP data. It is important to note that a traditional TRFLP approach was insufficient for this purpose due to the inability to effectively distinguish between the different distributions tested (Table 3). Applying the equations from Dunbar et al. [13] to a hypothetical community having the same structure of that observed in the GC-TRFLP data it can be estimated that 86,264 16S rRNA gene clones would need to be sequenced to recover the same 731 OTUs from this community with 95% confidence. Thus, the potential advantage of the GC-TRFLP approach is the ability to sample more taxa with less effort than would be required with other methods.

The well known hyper-diversity of soil microbial communities makes it important to consider the impact that under-sampling can have on the estimation of community SAD. The effect of under-sampling on estimates of community SAD was first explained by Preston who invoked the concept of the veil line [21]. When samples are acquired at random from the community the veil line can be understood as a veil curve and can impact the shape of the SAD [22]. Both GC-TRFLP and TRFLP sample a subset of the community but this subset is not drawn at random. Non-detection of rare taxa in these analyses results from failure of DNA templates to amplify in PCR when present below a threshold concentration. Thus, one would expect the result to be one of truncation of the SAD rather than a change in shape resulting from the effect of a veil curve. Since the fitting approach used in our study is concerned only with the ‘visible’ portion of the SAD it is not expected that this truncation should impact our ability to recapitulate community SAD.

To ensure that the flexibility of the lognormal is not the reason that it provides the best fit, we have used a method which is able to differentiate between a correct fit of the lognormal distribution to a community created with a lognormal distribution and an incorrect fit to various communities created with different distributions (Table 1). The ability to exclude potential distributions as that of the underlying community has great benefit even if the actual distribution cannot be conclusively identified. A re-sampling approach was used in place of maximum likelihood methods for fitting of distributions as the latter approach was deemed inappropriate for GC-TRFLP data. While GC-TRFLP decreases TRF overlap relative to TRFLP the possibility of different species with overlapping peaks still exists, thereby making it impossible to estimate OTU richness or mean abundance of OTUs in the true community and making traditional methods of maximum likelihood estimation less attractive as these values are required for most methods, and MLE would not account for any possibility of sequences from different sources overlapping within the same peak. The approach that was taken while unable to provide an estimate of richness for 16S rRNA based OTUs as conventionally defined (ie: at a 3% dissimilarity cutoff), nonetheless provides insight to the underlying form of the community distribution.

Differences were observed between electropherograms of T-RFLP and composite GC-TRFLP analyses of the same soil sample. This phenomenon likely results because DNA fractionation prior to PCR changes the outcome of DNA amplification. It is not accurate to describe this difference as a bias since the two methods would be expected a-priori to yield different results (and in fact this is why GC-TRFLP proves more useful than TRFLP in assessing community SAD). DNA composition and concentration has been shown to affect TRFLP profiles [23], and because DNA template composition and concentration necessarily varies between a bulk TRFLP analysis and twenty TRFLP analyses performed on the same DNA sample following fractionation, we would expect quantitative and qualitative differences in TRF detection.

Our simulations show that PCR bias, which results in changes in the relative abundance of OTUs, can alter the form of the community SAD. In the case of GC-TRFLP data, both Fisher's log-series and power-law communities were no longer recognized as such following simulated PCR bias (Table 4). The lognormal and geometric distributions fared somewhat better as the correct distributions were not rejected (Table 4). In addition, despite the PCR bias applied, the lognormal distribution was still correctly rejected for communities that had geometric or Fisher's log-series distributions (Table 4). It is interesting to note, however, that when PCR bias was applied to a community with a power-law distribution the lognormal distribution could no longer be rejected for that community (Table 4). As a result, though the GC-TRFLP data obtained for soil follows a lognormal distribution (Table 2) the potential for PCR bias means that we cannot completely reject the possibility that the true distribution of the soil community is a power-law function.

Other studies provide evidence that the SAD of soil bacteria could follow a power-law function. The Zipf distribution, a specific power-law function, was found to best describe the species abundance distribution of soil bacteria as calculated from DNA-DNA reassociation kinetics [11], although the mathematics used in these calculations has been contested by multiple groups [24], [25], [26], [27]. The Pareto distribution has also been considered for soil bacterial communities [28]. A generalized power-law function was chosen for the current analysis because both Zipf and Pareto distributions are specific instances of power-law functions which each contain only one parameter. The power-law function used in the present study has two parameters, is thus more flexible, and will encompass and fit well communities which possess either a Zipf or Pareto distribution.

Many different factors are involved in community formation and the abundance of species within those communities. One widely discussed and contested model is the neutral community model (NCM). The GC-TRFLP would potentially represent a useful way to assess the NCM, as the power of this model lies in its predictive power and a proper analysis of its validity would require comparing the SADs of multiple communities. A test of the NCM is beyond the scope of the current manuscript as such a test would require analysis of many samples to assess the distribution of the local community relative to that of the metacommunity. An assessment of this model for bacterial communities was recently performed using DGGE by Woodcock et al [29]. Another possible method for assessing the NCM is through pyrosequencing, though 50,000–100,000 reads would probably be necessary to sample community SAD with the same depth that is obtained through GC-TRFLP. While this approach would likely be fruitful, and would provide sequence information that would be far more useful than knowledge of TRF peaks, the advantage of the GC-TRFLP over sequencing approaches remains the issue of cost. GC-TRFLP provides far greater resolution of the community SAD than is possible with DGGE and allows for the processing of more environmental samples than could be currently achieved through sequencing approaches. Deciphering the effects of niche space, migration, resource availability, and the many other possible factors affecting SADs will require comparison of samples from many communities. We have found through probing a community of soil bacteria at unprecedented depth that, as with communities of larger organisms, the lognormal appears to be the appropriate null model for further investigations of bacterial SADs. Microbial communities could be ideal for the general study of SADs as well, as the degree of manipulation possible, speed and cost associated with a census of bacteria are far more amenable to testing a large number of hypotheses than with communities of plants or animals.

## Methods

### GC-TRFLP and single TRFLP

The GC-TRFLP dataset was generated to examine the microbial community of grass rhizosphere soil [30]. Soil was sampled from 0–5 cm depth in a fallow field in Ithaca NY that had not been cultivated for more than 30 years and is currently home to a diverse mix of perennial grasses and forbs. Five 2.5 cm diameter cores were randomly taken from a 1 m^2^ area, sieved to 4 mm and homogenized. DNA was extracted from four 0.25 g sub-samples of soil using the UltraClean Soil extraction kit (MoBio, Inc.) as per the manufacturer's instructions, and these DNA extracts were subsequently pooled. DNA was further purified by electrophoresis through a 1% agarose gel to remove fragments smaller than 4 kbp, DNA of greater than 4 kbp excised from the gel, agarose removed by digestion with agarase (New England Biolabs) as per the manufacturer's instructions, and DNA obtained by ethanol precipitation as described previously [31]. A total of 1.8 µg g^−1^ DNA was obtained, as determined by analysis of subsamples with the Quant-iT PICO Green dsDNA assay (Invitrogen) per the manufacturer's instructions.

CsCl gradient fractionation was carried out as described previously [32]. Briefly, primary CsCl gradients were formed by filling 4.7 ml polyallomer Optiseal tubes (Beckman) with 4.3 ml of gradient buffer (15 mM Tris-HCl, 15 mM KCl, 15 mM EDTA, pH 8.0) and 0.45 ml of DNA (1.8 µg) in TE buffer (50 mM Tris-HCl, 15 mM, pH 8.0) to obtain a homogeneous CsCl density of 1.69 g ml^−1^. Centrifugation was carried out for 66 h at 55,000 rpm (164,000×*g* maximum) at 20°C in an Optima Max-E tabletop centrifuge (Beckman-Coulter) equipped with a TLA110 rotor. A fraction recovery system (Beckman) was used to collect 45 fractions of 100 µl from the CsCl gradient, and the density of each fraction was determined by measurement of refractive index using an AR200 digital refractometer (Reichert).

CsCl was removed from DNA by ethanol precipitation, and DNA was resuspended in 25 µl of 50 mM Tris-HCl, pH 8.0, and stored at −20°C. DNA from gradient fractions was characterized by terminal restriction fragment length polymorphism (TRFLP) analysis of 16S rRNA genes. For both the GC-TRFLP and the single TRFLP, bacterial 16S rRNA genes were amplified by PCR using the primer Bact8F (5′-AGA GTT TGA TCM TGG CTC AG-3′), labeled at the 5′ end with the dye 6-carboxy-fluorescein, and the primer Univ1390R (5′-GAC GGG CGG TGT GTA CAA-3′). Reactions were carried out as described previously [32], PCR products were purified and resuspended in 50 mM Tris-HCl (pH 8.0), 250 to 400 ng of this DNA was digested with MspI (New England Biolabs) in 30-µl reaction volumes as per the manufacturer's instructions, and the enzyme was subsequently inactivated by incubation at 65°C for 20 min. The digested PCR products were desalted and concentrated again and then resolved on an Applied Biosystems Automated 3730 DNA analyzer.

### Creating artificial communities and in silico GC-TRFLP data

Artificial communities consisting of 16S rRNA sequences from the Ribosomal Database Project Release 9 [33] were generated by sampling individual sequences without replacement. RDP Release 9 was downloaded in June 2007, when there were 138,815 sequences over 1200 bp in length. The rank abundance of each sequence in the community was based on the order in which they were sampled. The abundance applied to each sequence was a function of either the lognormal, power-law, geometric, or Fisher's log-series distributions created with the parameters estimated from fitting the empirical data. GC-TRFLP data for these artificial communities was simulated in silico as follows. The mol% G+C content of the 16S rRNA sequence was used as a proxy for genome mol% G+C content to provide 20 bins simulating gradient fractionation of genomes by G+C content into 20 fractions. While 16S rRNA does not necessarily correlate strongly with 16S rRNA gene G+C content it does provide a convenient proxy which can be used to simulate the general effect of fractionating DNA by its G+C content. Restriction enzyme digestion of in silico community fractions was performed, simulating digestion with the restriction enzyme *MspI*. Sequences with TRF sizes greater than 550 bp were discarded because these fragments are generally not resolved in TRFLP. When two or more sequences with the same TRF size occurred in the same GC bin, the abundances of these sequences were summed. The community size varied based on the number of sequences required to reach 731 unique peaks, with 1041 sequences required on average for 100 artificial communities.

### Parameter estimation for actual and artificial communities

To find the distribution parameters that provide the best fit to GC-TRFLP data from actual and artificial communities, a SAD was simulated for a given distribution as described above and the parameters of the distribution were optimized by recursive iteration. The optimization process started by minimizing the χ^2^ value across all parameter combinations at a low resolution, to avoid falling in a valley of low χ^2^ values that did not contain the lowest value. The next step was to change each parameter individually, using the best value for each parameter over a range of values for the other parameters used in the distribution. This process was iterated until the χ^2^ value was minimized and the parameter estimates ceased to change. For example, if a distribution has two parameters, α and β, the distribution would be calculated and compared to the actual data for a range of α, holding β constant. Then the same would be done for β while holding α constant at the value which provided the best goodness-of-fit in the previous step. Once neither α and β changed in direct succession, they were assumed to be the best fitting parameters. To check this approach, the best fit was found by comparing all combinations of parameter values, and verifying that this matched the value found by the iterative approach. This comparison of all values was too computationally intensive to be used for all of the tests performed. As previously suggested [34], rank abundance data was not binned by abundance class to retain all possible information, allowing for more powerful hypothesis testing. For each set of parameters the χ^2^ value was calculated as an average from five different simulations.

### Estimating fit

Following the parameter estimation described above the estimate of fit for each distribution was evaluated by performing one hundred simulations with the best-fitting parameters and the distribution estimates were then compared to the actual or artificial distribution using the Kolmogorov-Smirnov (KS) test in the R software environment [35]. The KS test finds the maximum vertical distance between the empirical cumulative distribution functions of the two distributions in question. The values presented are an average of these one hundred measurements, referred to as the D-statistic, from which *p*-values are derived.

### Equations

The equations used for creating the distributions are as follows, each equation contained independent parameters that were estimated as previously described, in addition A_R_ is OTU abundance, and S_R_ is OTU rank:

Lognormal (independent parameters: α, μ, σ):
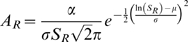

Power-law (independent parameters: α, β):
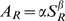

Geometric (independent parameters: α, β):
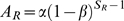

Fisher's Log-Series (independent parameters: α, β):
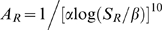

### PCR bias

As a guide for estimating PCR bias, we have used the study performed by Frey et al [20], in which changes in abundance due to PCR bias where measured when examining multitemplate communities with TRFLPs. We simulated PCR bias by creating biased original distributions through multiplying each value of the original distribution by 1.38*^r^*, where *r* is a random number between −1 and 1.
